# *Trypanosoma brucei* Glycogen Synthase Kinase-3, A Target for Anti-Trypanosomal Drug Development: A Public-Private Partnership to Identify Novel Leads

**DOI:** 10.1371/journal.pntd.0001017

**Published:** 2011-04-05

**Authors:** Richard O. Oduor, Kayode K. Ojo, Gareth P. Williams, Francois Bertelli, James Mills, Louis Maes, David C. Pryde, Tanya Parkinson, Wesley C. Van Voorhis, Tod P. Holler

**Affiliations:** 1 Opportunities for Partnering in Medicine, Pfizer Global Research and Development, Sandwich, Kent, United Kingdom; 2 Division of Allergy and Infectious Diseases, Department of Medicine, University of Washington, Seattle, Washington, United States of America; 3 High Throughput Screening Center of Emphasis, Pfizer Global Research and Development, Sandwich, Kent, United Kingdom; 4 Worldwide Medicinal Chemistry, Pfizer Global Research and Development, Sandwich, Kent, United Kingdom; 5 Laboratory for Microbiology, Parasitology and Hygiene, University of Antwerp, Antwerp, Belgium; New York University School of Medicine, United States of America

## Abstract

**Background:**

*Trypanosoma brucei,* the causative agent of Human African Trypanosomiasis (HAT), expresses two proteins with homology to human glycogen synthase kinase 3β (*Hs*GSK-3) designated *Tbru*GSK-3 short and *Tbru*GSK-3 long. *Tbru*GSK-3 short has previously been validated as a potential drug target and since this enzyme has also been pursued as a human drug target, a large number of inhibitors are available for screening against the parasite enzyme. A collaborative industrial/academic partnership facilitated by the World Health Organisation Tropical Diseases Research division (WHO TDR) was initiated to stimulate research aimed at identifying new drugs for treating HAT.

**Methodology/Principal Findings:**

A subset of over 16,000 inhibitors of *Hs*GSK-3 β from the Pfizer compound collection was screened against the shorter of two orthologues of *Tbru*GSK-3. The resulting active compounds were tested for selectivity versus *Hs*GSK-3β and a panel of human kinases, as well as *in vitro* anti-trypanosomal activity. Structural analysis of the human and trypanosomal enzymes was also performed.

**Conclusions/Significance:**

We identified potent and selective compounds representing potential attractive starting points for a drug discovery program. Structural analysis of the human and trypanosomal enzymes also revealed hypotheses for further improving selectivity of the compounds.

## Author Summary

Over 60 million people in sub-Saharan Africa are at risk of infection with the parasite *Trypanosoma brucei* which causes Human African Trypanosomiasis (HAT), also known as sleeping sickness. The disease results in systemic and neurological disability to its victims. At present, only four drugs are available for treatment of HAT. However, these drugs are expensive, limited in efficacy and are severely toxic, hence the need to develop new therapies. Previously, the short *Tbru*GSK-3 short has been validated as a potential target for developing new drugs against HAT. Because this enzyme has also been pursued as a drug target for other diseases, several inhibitors are available for screening against the parasite enzyme. Here we present the results of screening over 16,000 inhibitors of human GSK-3β (HsGSK-3) from the Pfizer compound collection against *Tbru*GSK-3 short. The resulting active compounds were tested for selectivity versus *Hs*GSK-3β and a panel of human kinases, as well as their ability to inhibit proliferation of the parasite *in vitro*. We have identified attractive compounds that now form potential starting points for drug discovery against HAT. This is an example of how a tripartite partnership involving pharmaceutical industries, academic institutions and non-government organisations such as WHO TDR, can stimulate research for neglected diseases.

## Introduction

Human African trypanosomiasis (HAT) and the lack of effective therapy constitute a health concern in 36 countries of sub-Saharan Africa [1]. The disease affects predominantly poor populations and transmission has been attributed to exposure during activities such as agriculture, animal husbandry, or hunting [2], which are the major means of livelihood in endemic regions. Following acute infection, the disease progresses to a chronic phase ultimately with invasion of the brain. This can happen within a month of initial infection, or alternatively can take years, depending on the parasite sub-species [3]. Four drugs, Eflornithine, Suramin, Pentamidine and Melarsoprol, are currently licensed for the treatment of HAT [4], [5]. Unfortunately, these are toxic and difficult to administer, limiting therapeutic choices [6]. Thus, new therapies for HAT are urgently needed.

Protein kinases, estimated to represent over 30% of all drug discovery programs, remain one of the most studied drug targets for a number of human and animal diseases [7]–[10]. More than 500 protein kinases have thus far been identified, many of which are linked to disease processes [11]. Of particular interest here is a serine/threonine glycogen synthase kinase -3 (GSK-3), which plays a role in the regulation of glycogen metabolism [12], [13], WNT signaling [14], cell cycle regulation [15], [16] and other processes. *Hs*GSK-3 has been investigated as a drug target for several diseases including Alzheimer's disease [17], neurodegeneration and oncogenesis [18]. Two isoforms of GSK-3 exist in human cells, *Hs*GSK-3 alpha and *Hs*GSK-3 beta. These human isoforms display a high degree of sequence identity with only one amino acid difference (Glu196 in *Hs*GSK-3 alpha and Asp133 in *Hs*GSK-3 beta) in the ATP binding domain [19], [20].

Previous studies [21] demonstrated that the causative agent of HAT, *Trypanosoma brucei*, expresses two proteins (*Tbru*GSK-3 short and *Tbru*GSK-3 long) with homology to *Hs*GSK-3. The shorter protein isoform was shown to be essential for parasite growth and viability and inhibitors of *Tbru*GSK-3 short were found to kill mammalian-stage *T. brucei*. The authors concluded that evolutionary variations in the ATP binding domain of *Tbru*GSK-3 short, relative to *Hs*GSK-3 beta, might allow for designing parasite selective inhibitors.

HAT drug development is challenged by the disproportionately small commercial interest and investment in developing new anti-parasite agents relative to other human diseases like cancer [22]. This study involved a collaborative Public-Private Partnership (PPP) facilitated by WHO TDR between researchers at University of Washington, USA, University of Antwerp, Belgium, and Pfizer Global Research Development, Sandwich, UK, to find specific inhibitors of *T.brucei* using a target-based high throughput screening (HTS) approach. This is an example of how drug development for neglected diseases can be stimulated by the PPP approach.

A panel of 16,540 putative inhibitors previously associated with projects at Pfizer targeting *Hs*GSK-3 was screened against the recombinant *Tbru*GSK-3 short. Selected hits were counter-screened against *Hs*GSK-3β. Kinase panel specificity and anti-parasitic screening were also conducted. Compounds identified in this study provide useful starting points for further chemical optimisation.

## Methods

### *T. brucei* GSK-3 short screening

Recombinant *Tbru*GSK-3 short (accession number Tb10.161.3140) was produced at the University of Washington [21] and shipped to Pfizer, Sandwich, UK for testing. Kinase-Glo reagent (Promega) was used as previously described [23]. This luciferase coupled assay, which provides a luminescent quantification of ATP consumed during the kinase reaction, was modified to a 384-well plate screening format. A selected library of 16,540 compounds comprising known *Hs*GSK-3 beta inhibitors and close structural analogues was screened at 10 µM final assay concentration. Assay plates were prepared by dispensing 0.2 µL of compound (dissolved in 100% DMSO) from master plates into white 384-well plates (Greiner bio one). Primary screening was conducted in a 20 µL reaction volume. Enzyme was added to each well to a final concentration of 3.8 nM in a volume of 10 µl using a Multidrop Combi dispenser (Thermo Scientific) and the plates were incubated for 15 minutes at room temperature (RT). Glycogen synthase peptide 2 (BioGSP2; Sigma) and ATP, were dissolved in 20% acetonitrile and 1 M Tris-HCl pH 7.6 respectively, then diluted in assay buffer to a final concentration of 3.2 µM BioGSP2 and 2 µM ATP. The assay buffer consisted of 25 mM Tris-HCl pH 7.5, 10 mM MgCl_2_, 5 mM DTT, 0.1 mg/mL BSA, 2 U/mL Heparin and 10 µM EDTA. The reaction was initiated by adding 10 µL of the substrate mixture to each well and allowed to proceed at RT for 2 h. Twenty microlitres of Kinase-Glo reagent was added to quench the reaction. Luminescence was measured after 1 h at a 100 millisecond/well integration time using the Acquest Multimode plate reader (Molecular Devices). Each plate included a positive control (4 µM GW8510, Sigma) and negative control (1% DMSO). Hit compounds were further titrated using a through-plate IC_50_ format with a maximum concentration of 25 µM. The data was analysed using Pfizer SIGHTS software and visualised using Spotfire software (TIBCO). Five separate 384-well plates were screened in duplicate to assess the assay reproducibility.

### Human GSK-3 beta screening

Human GSK-3 beta inhibition data (IC_50_) for many of the compounds were recovered from Pfizer data files. If historical data were not available, the compounds were tested in an assay using 10 nM *Hs*GSK-3 beta (Invitrogen) using Omnia Kinase Assay (Invitrogen) according to the manufacturer's instructions. The reaction volume was 20 µL and a range of compound concentrations were tested, up to a maximum of 40 µM. Briefly, 5 µl of *Hs*GSK-3 beta was dispensed into black 384 assay plates (Greiner bio one) containing 0.2 µl of compounds. The enzyme was incubated with the compounds for 15 minutes at 30°C then 15 µl of substrate mixture was added to each well to commence the reaction. The substrate mixture consisted of 2 µL each of 2× kinase reaction buffer, 10 µM Omina peptide substrate, 0.2 mM DTT and 10 µM ATP, and 7 µL of ultra pure water. The reaction was allowed to proceed for 30 minutes at 30°C. Increase in fluorescence levels indicating peptide phosphorylation by the enzyme was monitored using an Envision (PerkinElmer) with λex 360/λem 485 nm and the data were analysed using Pfizer software SIGHTS and Spotfire (TIBCO).

### Antiparasitic and kinase panel screening

Compounds with *Tbru*GSK-3 short IC_50_<100 nM were tested for their ability to inhibit the proliferation of *T. brucei* (blood stage form). Cytotoxicity testing against human fetal lung fibroblast MRC-5 cell line was also performed. Both assays were carried out with compound concentrations up to 64 µM at the Laboratory for Microbiology, Parasitology and Hygiene, University of Antwerp (www.ua.ac.be). Briefly, *T. brucei* trypomastigotes (Squib-427 strain, suramin-sensitive) were cultured in Hirumi-9 medium supplemented with 10% fetal calf serum at 1.5×10^4^ trypomastigotes per well. Following 72 hours incubation, parasite growth was assessed fluorimetrically by addition of resazurin. For cytotoxicity evaluation, 10^4^ MRC-5 cells/well were seeded onto the test plates containing the pre-diluted compounds and incubated at 37°C with 5% CO_2_ for 72 hours. Cell viability was determined fluorimetrically after addition of resazurin [24], [25]. Single point kinase panel screening was also conducted on selected compounds at 10 µM by Invitrogen (www.Invitrogen.com) and University of Dundee, UK (www.dundee.ac.uk).

### Modelling

The crystal structure of human GSK-3 beta complexed with staurosporine (pdb entry 1q3d) was used as the basis for modelling work. Selected compounds were docked into the crystal structure of *Hs*GSK-3 beta on the basis of binding modes of related known ligands. The binding-site residues were aligned and the residues that differ between human and *Tbru*GSK-3 in the catalytic pocket were highlighted with different colours. Images were created using the Pfizer molecule-modelling package MoViT.

## Results

### Assay Performance

**Figure 1:**
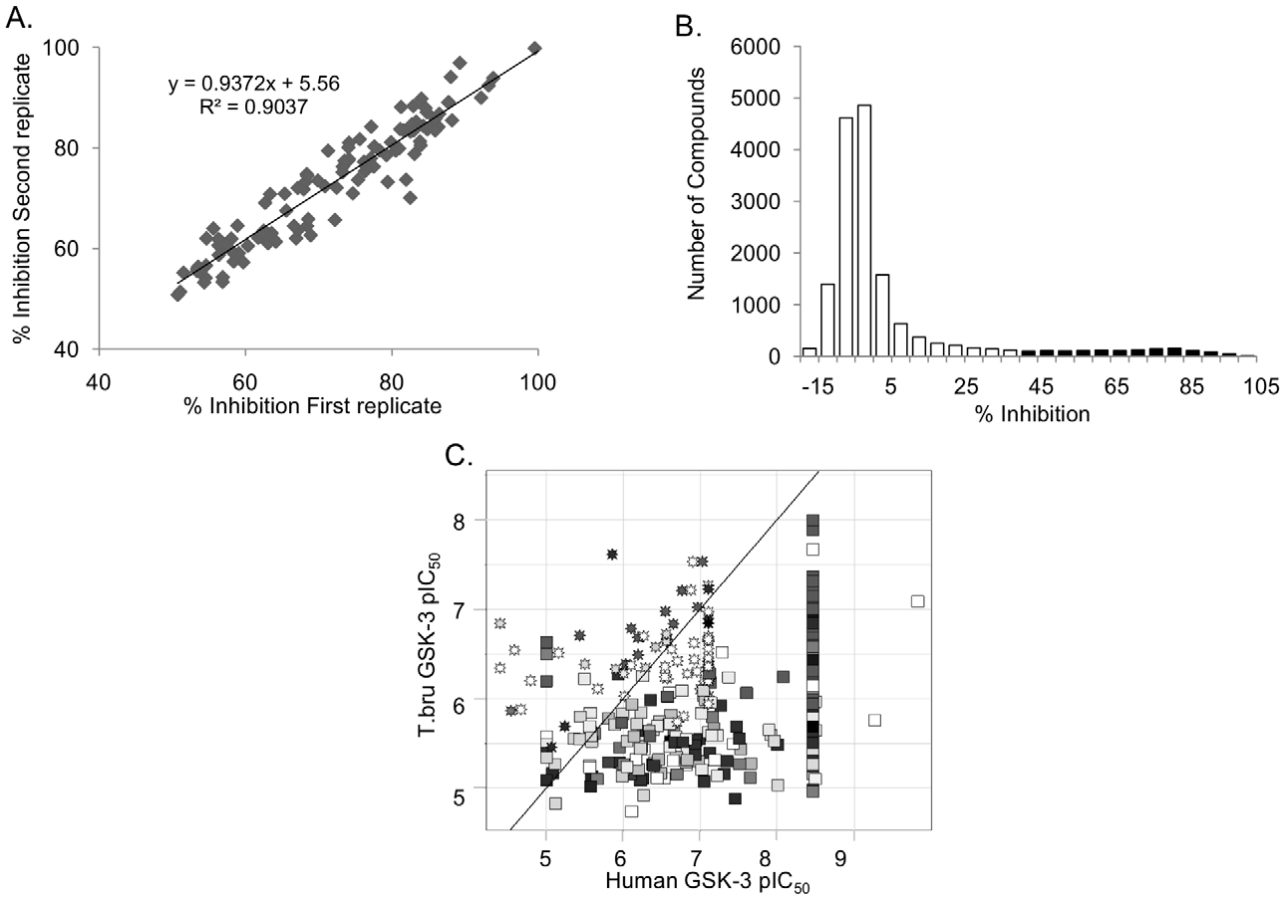
Screening results. (A) Verification of data reproducibility generated from duplicate testing of 5 separate 384-well plates. (B) Histogram plot showing distribution of hit compounds (black bars). (C) Plot of *Tbru*GSK-3 short pIC_50_ against *Hs*GSK-3 pIC_50_ revealing *Tbru*GSK-3 short selective compounds. Points are shaded by cluster ID and shaped by iteration, where squares are the original hits and stars are the near neighbours that were subsequently picked. pIC_50_ is -log IC_50_ where 9 = 1 nM and 6 = 1 µM.

A high throughput 384-well assay was developed for *Tbru* GSK-3 short which measures ATP depletion following phosphorylation of the peptide substrate BioGSP-2. The previously identified inhibitor of *Tbru*GSK-3 short, GW8510 [21], was used as a positive control. The assay yielded Z and Z' scores of 0.2 and 0.8, respectively, indicating excellent quality [26]. Assay reproducibility in HTS format was confirmed by duplicate testing of 5 separate 384-well plates which produced an identical number of hits (Figure 1A).

### GSK-3 Enzyme Screening

**Figure 2:**
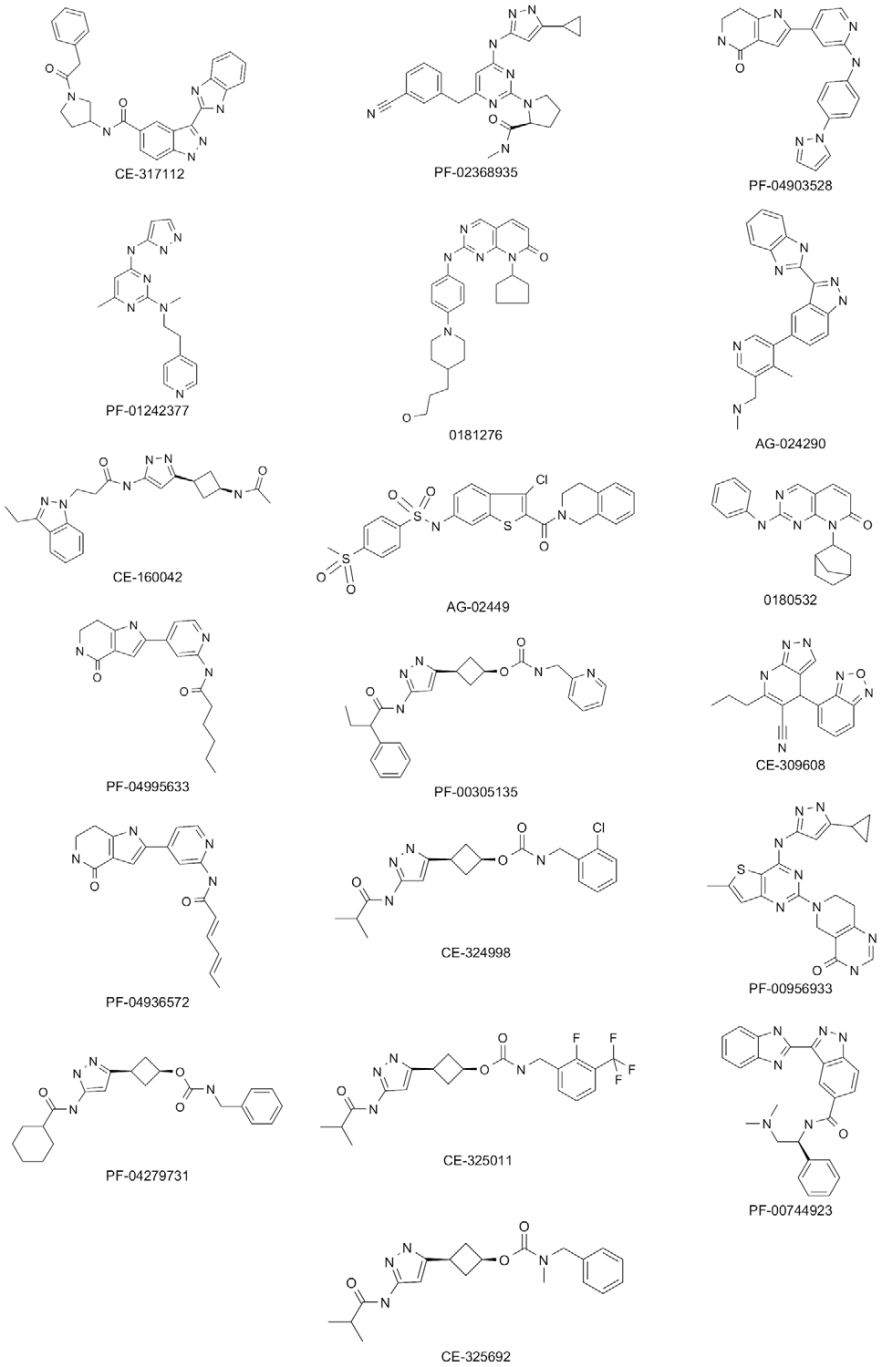
Chemical structures of the inhibitors.

A collection of 16,540 compounds targeting *Hs*GSK-3 beta were selected from Pfizer compound library and screened against *Tbru*GSK-3 short at a concentration of 10 µM. In order to capture all potential actives, compounds conferring above 40% inhibition were considered hits, giving an overall hit rate of 8.6% (Figure 1B). Hits were titrated in the screening assay, revealing 1,317 hits with IC_50_<25 µM. Of these confirmed hits, 362 compounds had IC_50_<1 µM and 35 compounds had IC_50_<100 nM. The IC_50_ data against *Hs*GSK-3 beta were either recovered from Pfizer records or the titration was conducted on selected compounds. A comparative analysis of inhibitor potencies between *Tbru*GSK-3 short and *Hs*GSK-3 beta is presented in Figure 1C. A majority of the compounds exhibited greater potency against the human enzyme which is not surprising, since the initial library was primarily made up of compounds that had been optimized for binding to *Hs*GSK-3 beta. Compounds were clustered with an in-house algorithm that carries out single-linkage clustering, whereby any pair of compounds sharing a Tanimoto similarity value of 0.7 (calculated using Daylight fingerprints) were placed in the same cluster (Daylight Chemical Information System Inc). These hits were expanded by selecting near neighbour analogues from the Pfizer compound libraries and further titrating them against both *Hs*GSK-3 beta and *Tbru*GSK-3 short. Two compounds, 0181276 and PF-04903528, were found to show 7-fold selective inhibition of *Tbru*GSK-3 short compared to *Hs*GSK-3 beta (Table 1 and Figure 2).

**Table 1:** GSK-3 Enzyme, Antiparasitic and cytotoxicity testing (values in µM).

Compound ID	*Tbru* GSK-3 short IC_50_	*HS*GSK-3 IC_50_	*Tbru* parasite EC_50_	MRC-5 EC_50_
CE-317112	0.099	0.003	1.14	39.40
PF-01242377	0.061	0.132	0.13	1.18
CE-160042	0.142	0.032	>25	NT
PF-04995633	0.097	NT	0.65	0.82
PF-04936572	0.091	NT	0.21	0.23
CE-325692	0.065	0.002	0.64	4.67
PF-00744923	0.070	<1.000*	0.13	0.13
AG-02449	0.069	NT	0.13	0.62
PF-04903528	0.032	0.236	1.13	1.13
PF-02368935	0.019	<1.000*	0.13	0.13
PF-00305135	0.067	0.003	2.45	0.93
CE-309608	0.021	0.046	2.69	16.00
PF-00956933	0.057	<1.000*	0.13	2.17
AG-024290	0.062	0.173	0.13	0.13
PF-04279731	0.062	0.001	1.23	1.47
CE-324998	0.093	0.001	0.23	1.22
CE-325011	0.094	0.005	1.05	2.32
0181276	0.600	4.29	NT	NT
0180532	7.27	0.06	NT	NT

Values are a mean of at least 2 replicates. NT  =  not tested due to limited compound availability.

*Due to limited compound availability, these compounds were only tested at a single concentration of 1 µM and showed >50% inhibition at this concentration.

### Whole Parasite Screening

Seventeen compounds with *Tbru*GSK-3 short IC_50_ values of <100 nM (regardless of selectivity) were tested for their ability to inhibit the proliferation of mammalian-stage *T. brucei.* Specificity for the parasite was investigated by testing against the human fetal lung fibroblast MRC-5 cell line (Table 1 and Figure 2). Ten compounds showed *in-vitro* inhibition of *T. brucei* proliferation with EC_50_s of <1 µM and 6 had EC_50_s of 1–3 µM. Several of the most potent compounds also showed potent inhibition of the MRC5 cell line. However, six compounds showed at least a 5-fold window between *T. brucei* activity and activity on MRC5 cells, particularly CE-317112 which had 35-fold selectivity (Table 1). In general, potent inhibition of *Tbru*GSK-3 enzyme activity correlated with potent activity against the whole parasite. However, CE-160042 which was a potent inhibitor of *Tbru*GSK-3 enzyme activity, showed no inhibition of the whole parasite (EC_50_ >25 µM). We subsequently discovered that this compound showed no detectable cell permeability in a standard CaCo2 cell flux assay used routinely in drug discovery (data not shown) and therefore the lack of activity is most likely due to the compound failing to reach the target within the parasite.

### Human Kinase Specificity

**Figure 3:**
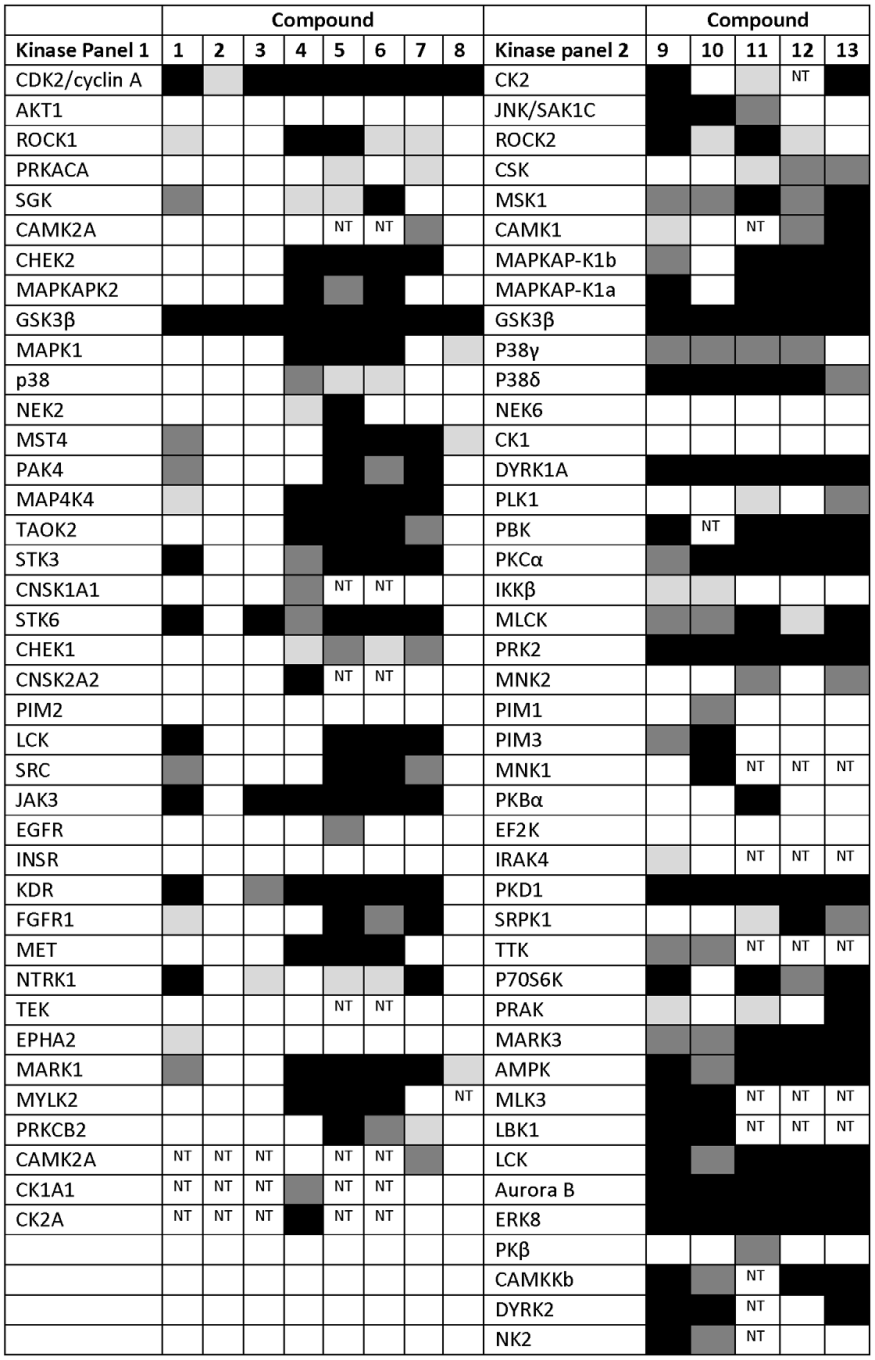
Kinase selectivity screening. Compounds were screened in one of two kinase panels at a concentration of 10 µM. The degree of inhibition of each kinase is indicated by shading as follows: white <29% inhibition, light grey 30-49% inhibition, dark grey 50-69% inhibition, black >70% inhibition. Key to compounds: (1) 0181276, (2) CE-160042, (3) 0180532, (4) PF-4903528, (5) PF-4936572, (6) PF-4995633, (7) AG-24290, (8) PF-4279731, (9) CE-317112, (10) PF-1242377, (11) PF-744923, (12) PF-2368935, (13) PF-956933. NT  =  not tested.

Human kinase inhibitors often inhibit more than one kinase leading to safety issues. In order to understand the kinase inhibition profile of *Tbru*GSK-3 inhibitors, 13 of the compounds were screened at 10 µM against a panel of approximately 40 human kinases. One of the compounds, CE-160042, was highly specific and only inhibited *Hs*GSK-3 beta (Figure 3). PF-4279731 and 0180532 were also relatively specific showing >50% inhibition of only 2 and 4 other kinases, respectively. The remaining compounds were active against more than 10 other kinases.

### Modelling

**Figure 4:**
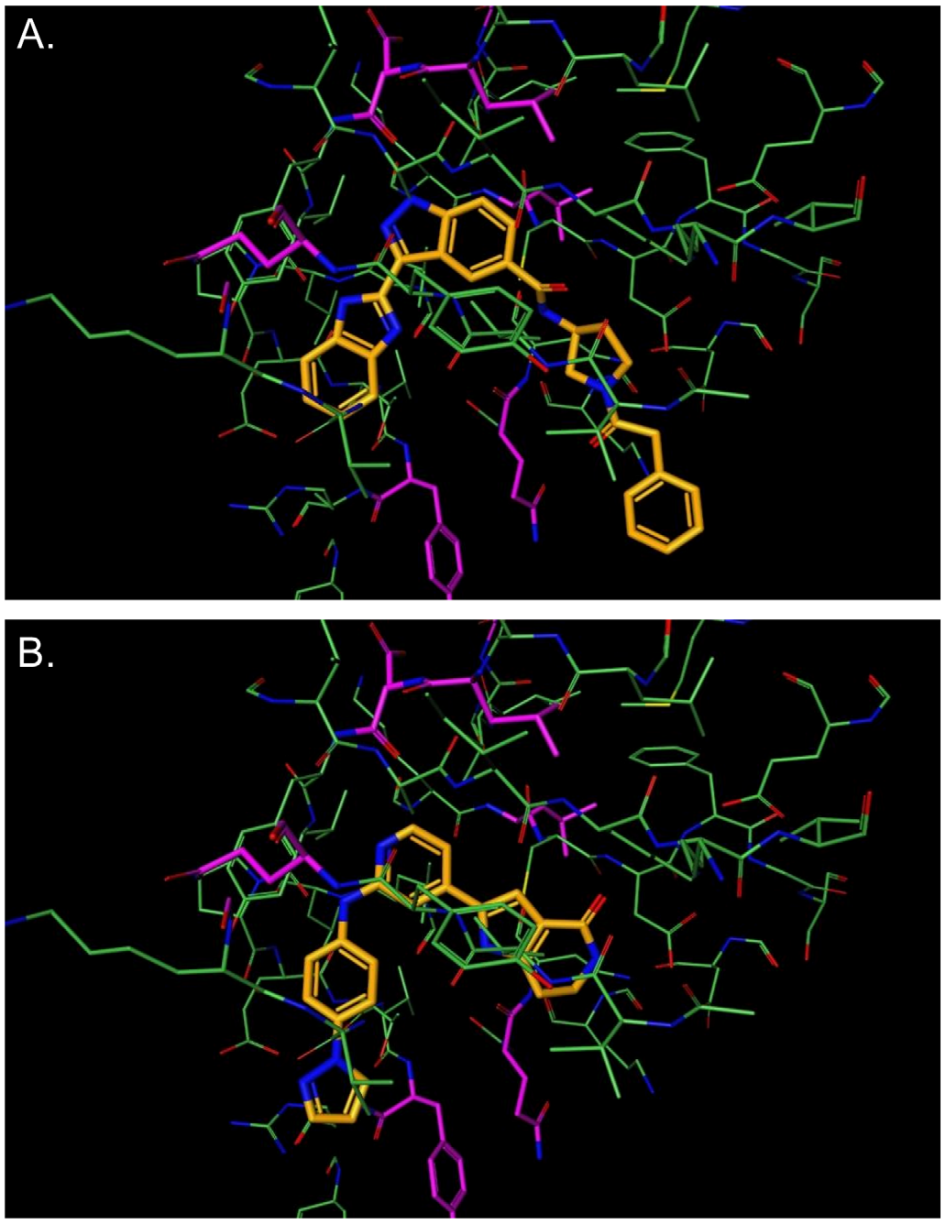
Modelling of the binding-site residues with putative inhibitors. Compounds (orange) docked into the catalytic domain of the crystal structure of *Hs*GSK3 beta in their binding modes. A: CE-317112 shows preference for *Hs*GSK-3 beta. B: PF-4903528 shows preference for *Tbru*GSK-3 short. The residues that differ between human and *Tbru*GSK-3 short are shown in magenta, with only L132M (top centre of the image) directly lining the pocket. Images were created using the Pfizer molecule-modelling package MoViT.

Previous modelling of the *Tbru* GSK-3 active site identified a number of residues that differ between the human and parasite enzyme that could potentially be exploited to achieve selective inhibition. Using the published enzyme structures [21], the predicted binding modes of two of our compounds were examined (Figure 4). This demonstrated that of the previously reported binding site differences, only one, *T.bru* M101/*Hs* L132 is in close proximity to the compound binding site and therefore is likely to be the key residue for achieving selectivity. The modelling suggests that greater selectivity could be achieved by making compounds with substituents that have improved interaction with methionine compared to leucine at this position.

## Discussion

We have exploited knowledge of the essentiality of *Tbru*GSK-3 short and the availability of a large number of *Hs*GSK-3β inhibitors to initiate a drug discovery program for Human African Trypanosomiasis. Over 16,000 compounds were screened against *Tbru*GSK-3 short isoform and compounds of interest were tested against *Hs*GSK-3 beta, whole parasites and human cells. Specificity against a panel of approximately 40 human kinases was also evaluated. We identified 2 compounds with approximately 7-fold selectivity for *Tbru*GSK-3 short over *Hs*GSK-3 beta: PF-04903528 and 0181276. One of these, 0181276 was also relatively specific against the wider human kinase panel. CE-160042 was not selective against the parasite enzyme, but was completely selective for *Hs*GSK-3 beta and showed no significant inhibition of any other kinases. In addition, CE-317112 showed a 35-fold safety window relative to the cytotoxicity control. Together, these compounds represent an attractive starting point for medicinal chemistry with a focus on further improving selectivity for a drug discovery program.

Using structural modelling, we have shown that improved selectivity may be possible by exploiting the *T.bru* M101/Hs L132 active site difference. Given that this is a relatively small difference, highly selective compounds may be difficult to obtain, however it is encouraging that our intitial screening has identified compounds with 7-fold selectivity. Previous studies suggest that *in vivo* inhibition of mammalian GSK-3 causes no significant changes in body weight, food consumption or any associated adverse effects, as judged by histopathology or blood chemistry analyses [27], [28]. Therefore, low levels of specificity may be tolerated. However, mouse knock-out studies of GSK-3 beta have shown embryonic lethality due to liver degeneration and changes in bone development [29], [30]. Consequently, non-selective inhibitors would not be safe for use in pregnant women, infants and young children. Therefore, selective inhibitors of the parasite enzyme would be highly desirable and the availability of the GSK-3 structural models provides a powerful tool for structure assisted compound design which could guide synthesis of more selective compounds, based on the initial 7-fold selective compounds we have identified.

This early drug discovery collaboration was facilitated by WHO TDR and demonstrates the power of such public private partnerships in bringing together the drug discovery expertise of pharma companies, the detailed target knowledge from academia and access to parasite biological assays from expert screening centers to accelerate drug discovery for neglected tropical diseases. Our most promising compounds are disclosed to accelerate the pace of drug development for HAT.

## References

[1] Bouteille B, Oukem O, Bisser S, Dumas M (2003). Treatment perspectives for human African trypanosomiasis.. Fundam Clin Pharmacol.

[2] Simarro PP, Jannin J, Cattand P (2008). Eliminating human African trypanosomiasis: where do we stand and what comes next?. PLoS Med.

[3] Burchmore R J, Ogbunude PO, Enanga B, Barrett MP (2002). Chemotherapy of human African trypanosomiasis.. Curr Pharm Des.

[4] Pepin J, Milord F (1994). The treatment of human African trypanosomiasis.. Adv Parasitol.

[5] Kennedy PG (2008). The continuing problem of human African trypanosomiasis (sleeping sickness).. Ann Neurol.

[6] Rodgers J, Stone TW, Barrett MP, Bradley B, Kennedy PG (2009). Kynurenine pathway inhibition reduces central nervous system inflammation in a model of human African trypanosomiasis.. Brain.

[7] Naula C, Parsons M, Mottram JC (2005). Protein kinases as drug targets in trypanosomes and Leishmania.. Biochim Biophys Acta.

[8] Doerig C (2004). Protein kinases as targets for anti-parasitic chemotherapy.. Biochim Biophys Acta.

[9] Weinmann H, Metternich R (2005). Drug discovery process for kinase inhibitors.. Chembiochem.

[10] Card A, Caldwell C, Min H, Lokchander B, Hualin X (2009). High-throughput biochemical kinase selectivity assays: panel development and screening applications.. J Biomol Screen.

[11] Manning G, Whyte DB, Martinez R, Hunter T, Sudarsanam S (2002). The protein kinase complement of the human genome.. Science.

[12] Doble BW, Woodgett JR (2003). GSK-3: tricks of the trade for a multi-tasking kinase.. J Cell Sci.

[13] Jope RS, Johnson GVW (2004). The glamour and gloom of glycogen synthase kinase-3 Trends Biochem.. Sci.

[14] Willert K, Nusse R (1998). β-catenin: a key mediator of Wnt Signalling.. Curr Opin Genet Dev.

[15] Diehl JA, Cheng M, Roussel MF, Sherr CJ (1998). Glycogen synthase kinase-3β regulates cyclic D1 proteolysis and subcelullar localization.. Genes Dev.

[16] Yost C, Torres M, Miller JR, Huang E, Kimelman D (1996). The axis-inducing activity, stability, and subcellular distribution of beta-catenin is regulated in Xenopus embryos by glycogen synthase kinase 3.. Genes Dev.

[17] Leclerc S, Garnier M, Hoessel R, Marko D, Bibb JA (2001). Indirubins Inhibit Glycogen Synthase Kinase-3β and CDK5/P25, Two Protein Kinases Involved in Abnormal Tau Phosphorylation in Alzheimer's Disease.. The Journal of Biological Chemistry.

[18] Martinez A, Castro A, Dorronsoro I, Alonso M (2002). Glycogen synthase kinase 3 (GSK-3) inhibitors as new promising drugs for diabetes, neurodegeneration, cancer, and inflammation.. Med Res Rev.

[19] Bertrand JA, Thieffine S, Vulpetti A, Cristiani C, Valsasina B (2003). Structural characterization of the GSK-3beta active site using selective and non-selective ATP-mimetic inhibitors.. J Mol Biol.

[20] Liao JJ (2007). Molecular recognition of protein kinase binding pockets for design of potent and selective kinase inhibitors.. J Med Chem.

[21] Ojo KK, Gillespie JR, Riechers AJ, Napuli AJ, Verlinde CL (2008). Glycogen synthase kinase 3 is a potential drug target for African trypanosomiasis therapy.. Antimicrob Agents Chemother.

[22] Trouiller P, Olliaro P, Torreele E, Orbinski J, Laing R (2002). Drug development for neglected diseases: a deficient market and a public-health policy failure.. Lancet.

[23] Ojo KK, Larson ET, Keyloun KR, Castaneda LJ, Derocher AE (2010). Toxoplasma gondii calcium-dependent protein kinase 1 is a target for selective kinase inhibitors.. Nature Structural & Molecular Biology.

[24] Cos P, Vlietinck AJ, Berghe DV, Maes L (2006). Anti-infective potential of natural products: How to develop a stronger in vitro ‘proof-of-concept’.. Journal of Ethnopharmacology.

[25] Baldé ES, Megalizzi V, Traoré MS, Cos P, Maes L (2010). In vitro antiprotozoal, antimicrobial and antitumor activity *of* Pavetta crassipes K. Schum leaf extracts.. Journal of Ethnopharmacology.

[26] Zhang J, Chung TDY, Oldenburg KR (1999). A Simple Statistical Parameter for Use in Evaluation and Validation of High Throughput Screening Assays.. J Biomol Screen.

[27] Henriksen EJ, Kinnick TR, Teachey MK, O'Keefe MP, Ring D (2003). Modulation of muscle insulin resistance by selective inhibition of GSK-3 in Zucker diabetic fatty rats.. Am J Physiol Endocrinol Metab.

[28] Kaidanovich-Beilin O, Eldar-Finkelman H (2006). Long-term treatment with novel glycogen synthase kinase-3 inhibitor improves glucose homeostasis in ob/ob mice: molecular characterization in liver and muscle.. J Pharmacol Exp Ther.

[29] Kugimiya F, Kawaguchi H, Ohba S, Kawamura N, Hirata M (2007). GSK-3b Controls Osteogenesis through Regulating Runx2 Activity.. PLoS ONE.

[30] Hoeflich KP, Luo J, Rubie EA, Tsao M, Jin O (2000). Requirement for glycogen synthase kinase-3β in cell survival and NF-kβ activation.. Nature.

